# Exploration on intelligent control of the hospital infection -the intelligent reminding and administration of hand hygiene based on the technologies of internet of things

**DOI:** 10.1186/1479-5876-10-S2-A55

**Published:** 2012-10-17

**Authors:** Jieran Shi, Lize Xiong, Shengxing Li, Hua Tian

**Affiliations:** 1 The First Affiliated Hospital of the Fourth Military Medical University, Xi’an, Shanxi, China; 2 Zhejiang Enjoyor Electronics Co., Ltd., Hangzhou, Zhejiang, China; 3 Zhejiang Association of IOT Industry, Hangzhou, Zhejiang, China

## 

The administration and control of the hospital infection is one of the most important parts in the quality management of modern hospital and medical security. The hand hygiene is a major subject in the administration and control of the hospital infection. As evidences show, the hands of the medical staff carry a lot of germs and up to 30 percents of the hospital infection result from the situation that the medical staff does not wash their hands according to the desired standard. Normative hand hygiene can make the hospital infection rate reduced by 50 percents.

There are several factors that affect the compliance of the hand hygiene of the medical staff. How to improve the poor compliance is a very challenging problem in the administration and control of the hospital infection. The developments of intelligent reminding and administration of hand hygiene system (IRAHHS) is a good way to radically solve the problem.

Based on the Radio Frequency Identification technology and intelligent analysis system, the IRAHHS has many reading and writing devices with perception in major infection control areas, danger areas such as around infected articles and the bedside of the patients, and hand disinfect or washing areas. When the medical staff with intelligent electronic tags touches the sources of the pollution in these areas, the electronic tags communicate with the devices and record and remind the staff of the status of their hand hygiene and ask them to do required hand hygiene behaviors. The information will be recorded and sent to the backstage simultaneously. The IRAHHS is connected to the central information system of the hospital and provides the manager with the ability to search, access, analyze the data and judge.

The IRAHHS achieves the functions of intelligent perception, reminding and tracking with the help of technologies of Internet of Things. It is a novel attempt in the administration of hand hygiene and also a new idea in the administration and control of the hospital infection in China.

